# Influence of Porosity on the Morpho-Structure, Physical-Chemical and Biochemical Characteristics of Polylactic Acid and/or Polycaprolactone Scaffolds

**DOI:** 10.3390/polym17172311

**Published:** 2025-08-26

**Authors:** Anca Peter, Manuel Brendon Monea, Anca Mihaly Cozmuta, Camelia Nicula, Leonard Mihaly Cozmuta, Zorica Vosgan, Zsolt Szakacs, Goran Drazic, Klara Magyari, Marieta Muresan-Pop, Lucian Baia

**Affiliations:** 1 Faculty of Sciences, Technical University of Cluj Napoca, Victoriei 76, 430072 Baia Mare, Romania; manumonea77@gmail.com (M.B.M.); mihaela.mihaly@cb.utcluj.ro (A.M.C.); camelia.nicula@cb.utcluj.ro (C.N.); leonard.mihaly@cb.utcluj.ro (L.M.C.); zorica.vosgan@cb.utcluj.ro (Z.V.); zsolt.szakacs@econ.utcluj.ro (Z.S.); 2 National Institute of Chemistry, Hajdrihova 19, P.O. Box 660, SI-1001 Ljubljana, Slovenia; goran.drazic@gmail.com; 3 Interdisciplinary Research Institute on Bio-Nano-Sciences, Nanostructured Materials and Bio-Nano-Interface Center, Babes-Bolyai University, 42, Treboniu Laurian, 400271 Cluj-Napoca, Romania; klara.magyari@ubbcluj.ro (K.M.); marieta.muresan@ubbcluj.ro (M.M.-P.); lucian.baia@ubbcluj.ro (L.B.); 4 INSPIRE Research Platform, Babes Bolyai University, 11 Arany Janos Street, 400084 Cluj-Napoca, Romania; 5 Faculty of Physics, Babes-Bolyai University, M. Kogălniceanu 1, 400084 Cluj-Napoca, Romania; 6 Institute for Research-Development-Innovation in Applied Natural Sciences, Babes-Bolyai University, Fântânele 30, 400294 Cluj-Napoca, Romania

**Keywords:** polylactic acid, polycaprolactone, porosity, crystallinity, thermostability, mechanical resistance, nutrients uptake, hydrophilicity, biodegradability, microbiological biocompatibility

## Abstract

The design and development of scaffolds play a crucial role in tissue engineering. In this regard, the study aims to establish the influence of porosity on the morpho-structural, physical–chemical, and biochemical characteristics of the polylactic acid (PLA) and/or polycaprolactone (PCL) scaffolds, in order to be considered candidates for tissue reconstruction. The results indicated that binary PLA-PCL and PCL matrices are more suitable than PLA, due to their higher crystallization degree, this contributing to the superior mechanical properties and lower network defects. The preponderance of molecular interactions decreases with porosity. Porosity induced a decrease in the degree of crystallization of PLA-PCL and an increase in water, glucose and blood components uptake by 188, 178, and 28%, respectively. The PLA-PCL scaffold was found to be more stable to lipase action than neat PLA as a result of the reduced enzyme access due to the higher crystallinity and thermodynamic stability of the hydrocarbon linear chain in PCL, which is higher than that of the side methyl group in PLA. *Lactobacillus* growth increases with porosity and was more pronounced on the PLA-PCL matrix. All these results show that varying the porosity and composition of the polymer mixture leads to valuable materials with nutrient absorption capacity and biodegradability superior to neat PLA or PCL materials.

## 1. Introduction

The design and development of scaffolds play a crucial role in tissue engineering. Their characteristics are essential for functioning as synthetic extracellular matrices [1]. A scaffold for tissue development may be partly viewed as an engineering challenge, in which a multiscale list of key requirements must be fulfilled to achieve an optimal process. Thus, the matrix must have mechanical strength, high porosity, and large interconnected pores facilitating cell development and the circulation of nutrients and residues. Also, morphology is essential in creating surfaces conducive to cell adhesion. The ideal range varies depending on the tissue type, and 200–250 μm is a recommended range for certain soft tissues [2]. In addition, the absorption capacity of organic matter (carbohydrates and protein), as well as the resistance to enzymatic action are important [3]. In the literature, a large variety of materials have been proposed for ligament scaffolds, including polyetheretherketone (PEEK), polylactic acid (PLA), polyglycolic acid (PGA), polycaprolactone (PCL), and copolymers based on them, such as copolymer PLA-PGA (PLGA) and copolymer PLA-PCL (PLCL), for their biodegradability and material characteristics [2]. PCL is intensively used for tissue reconstruction due to its biocompatibility. Its hydrophobic character may limit cell adhesion, but this disadvantage could be overcome by surface modification [3]. PCL is a thermoplastic polymer with a low melting point and high elasticity, but with low mechanical and thermal resistance. In this sense, combining it with other types of biomaterials could be a solution to improve these drawbacks, thus achieving the ideal characteristics of a scaffold for tissue reconstruction [4]. PLA is one of the most compatible thermoplastics with scaffolding potential for tissue reconstruction. It has mechanical resistance and stability even at higher temperatures [3]. Kumar et al. [3] developed PCL-PLA matrices by porogen leaching, considered the most acceptable composites for tissue reconstruction, in the form of interspersed PCL-PLA domains. The results showed that the mechanical properties, adhesion, and cell proliferation were superior in the case of the PLA-PCL composite than for neat PCL. Other studies have reported successful testing of the PLA-PCL compound as a stent [5] or as a bone substitute in dogs, following association with hydroxyapatite [6]. Apart from soft tissue reconstruction, PCL is also studied as a substitute biomaterial for other organs, such as the dermis [7], nerves [8], or myocardium [9]. Chen et al. [7] reported a fetal dermis-inspired architectural scaffold designed for scarless dermal regeneration with polycaprolactone/collagen/hyaluronic acid, fabricated successively through porogen-leaching and electrospinning methods, demonstrating desirable hydrophilic properties and wettability, fibroblast proliferation, and no excessive inflammatory reaction, denoting good biocompatibility and a prospect of tissue engineering in vivo. Rejali et al. [9] developed polyglycerol sebacate/polycaprolactone/reduced graphene oxide composite scaffolds for myocardial tissue engineering and they proved that adding 2%wt reduced graphene oxide to the composite scaffold decreased the diameter and degradation rate while increasing electrical conductivity and ductility. Mechanical evaluations revealed results close to the heart muscle’s elastomeric properties. Soltani Gerdefaramarzi et al. [8] tested the PLA-PCL–graphene composite as a potential candidate for the reconstruction of the nerve tissue. They showed that a composite scaffold containing 50 wt% PLA, 48.5 wt% PCL, and 1.5 wt% graphene satisfies the requirements for the peripheral nerve tissue. Eryildiz et al. [10] showed that the addition of 20 wt% PCL resulted in PLA/PCL blend scaffolds with improved mechanical and thermal properties, showing promise for future tissue engineering applications. Solechan et al. [11] produced PLA/PCL blend by ball milling, followed by pressure compaction and sintering and their work revealed a number of modifications in the functional groups and crystal phase. They also showed that the porosity decreased with increasing PLA concentration so that the density and flexural properties of the PLA/PCL blend increased.

Porogen leaching is one of the oldest technologies for porous product development. It is based on dispersing a template within a polymeric solution, gelling, and removing the template to result in a porous morphology. Porogen leaching applies to various polymers, and the dissolution of “sacrificial structures” is a common feature in many fabrication approaches for vascularization and tissue reconstruction [12]. Yildirim et al. [1] combined porogen leaching and supercritical drying with liquid CO_2_ to prepare polycaprolactone/graphene oxide microporous interconnected structures and showed that PCL–graphene oxide scaffolds do not possess any toxicity towards L-929 mouse fibroblasts. In addition to classical techniques, such as porogen leaching, advanced methods, like melt electro-writing, have been explored for the fabrication of PLA, PCL, and their composite scaffolds, enabling highly organized structures with mechanical improvements for tissue engineering applications [13].

Another study [14] evaluated a multilayer electrospun scaffold composed of polyurethane (PU), platelet-derived growth factor (PRGF), and gelatin, on which human mesenchymal stem cells, fibroblasts, and *L. plantarum* were cultured. The results showed that the presence of *L. plantarum* had a positive effect on cell viability and increased the activity of genes involved in fibroblast migration and proliferation. Although there are no specific studies on the direct interaction between PLA/PCL scaffolds fabricated by porogen leaching and *L. plantarum*, the combination of these elements could be explored for the development of biomaterials with biocompatibility and probiotic properties. The incorporation of *L*. *plantarum* into PLA/PCL scaffolds could offer benefits in infection prevention and tissue regeneration.

After a literature review, it was found that information about the influence of porosity on morpho-structure mechanical strength, thermostability, nutrient absorption, biodegradability, hydrophilicity, and microbiological biocompatibility of PLA and/or PCL-based materials is very limited. In this regard, the purpose of this study is to establish the influence of porosity on the abovementioned properties, which are considered essential factors for a biomaterial to be considered for tissue reconstruction.

## 2. Materials and Methods

### 2.1. Reagents

PLA was purchased from Nature Works (Minnetonka, MN, USA). Dichloromethane, silver nitrate, monopotassium phosphate, disodium phosphate, and ethanol (96%) were purchased from Chemical Company (Iași, Romania). Sodium chloride, pancreatic lipase, and glucose were purchased from Merck (Darmstadt, Germany). Albumin and the reagents used for glucose and albumin analysis were purchased from Biosystem (Bucharest, Romania). The microbiological biocompatibility evaluation of the scaffolds was performed using a probiotic powder containing viable *Lactobacillus plantarum* (Swanson^®^, Fargo, ND, USA).

### 2.2. Preparation of Scaffolds

The precursors and quantities used to prepare the scaffolds are presented in Table 1. PLA is of the Ingeo™ Biopolymer 4043D type. PCL is poly(ε-caprolactone), with an Mn of 80,000. The scaffolds were prepared by porogen leaching, using sodium chloride as porogen, according to the procedure described by Yildirim et al. [1] with some changes. The amounts of PLA and/or PCL indicated in Table 1 were mixed with the dichloromethane at room temperature under magnetic stirring (250 rpm), until complete dissolution of the PLA and/or PCL granules, respectively. Then, sodium chloride granules were added, followed by manual homogenization for 2 min and distribution into Teflon vessels with a 2 cm diameter. The samples were dried in air for 48 h and then washed with distilled water until no AgCl precipitate formed upon the addition of silver nitrate solution 5% (wt), indicating that NaCl was successfully removed (Table 1). Then, the samples were allowed to dry at 40 °C for 36 h in the oven.

### 2.3. Characterization of Scaffolds

#### 2.3.1. Morphology and Elemental Analysis

The scaffolds’ morphology was determined by optical and scanning electron microscopy (SEM).

A BRESSER LCD Micro Microscope with a magnification 10× was used to collect the micro-images.

The SEM images were recorded with a Jeol ARM 200 CF SEM (JEOL Ltd., Tokyo, Japan) Cs microscope operating at a 10 kV accelerating voltage with a working distance of 8 mm. A secondary electron (SE) detector was employed to capture surface morphology. Samples were sputter-coated with a thin layer of carbon to improve conductivity. Elemental analysis was determined by energy-dispersive X-ray spectrometry (EDXS). An energy-dispersive X-ray spectrometer (SDD) (model: Jeol Centurion) was used. The working potential was set to 30 kV.

#### 2.3.2. Structure

The scaffolds’ structure was investigated by X-ray diffraction (XRD) and Fourier transform infrared spectroscopy (FTIR).

A Shimadzu X-ray diffractometer (XRD 6000, Kyoto, Japan) operated with Cu-Kα radiation (λ = 1.54 Å) and a Ni filter was used to investigate the crystallization degree of the scaffolds. The diffraction patterns were recorded in a 2θ range from 10° to 80° with a scan speed of 2°/min. The crystallinity rate of the sample was calculated with the following equation:

(1) $$Degree\;of\;crystallinity\;(\%)=\;\frac{I_{crystalline}}{I_{total}}\times 100$$

where the *I_crystalline_* and *I_total_* were calculated by using WinPLOTR software 2019 included in FullProf_Suite. The primary crystallite size of the materials was calculated using the Scherrer equation.

A Perkin Elmer Spectrum BX spectrometer (PerkinElmer Inc., Waltham, MA, USA) was used to collect the FTIR spectra. The wavenumber ranged from 4000 to 600 cm^−1^. Each FTIR spectrum is the average of 8 measurements. The resolution was 4 cm^−1^. The transmittance values from the FTIR spectra were standardized using the following formula in order to be able to compare the results: standardized value = (experimental value − average value)/standard deviation.

#### 2.3.3. Differential Thermal Analysis (DTA) and Thermogravimetry (TGA)

For thermal and thermogravimetric measurements, a DTG-60H Derivatograph (Shimadzu Corporation, Kyoto, Japan) capable of recording simultaneously the differential thermal analysis (DTA) curve and the thermogravimetric (TGA) curve was used. The sample support was an alumina crucible covered with a perforated alumina lid, and the reference material was α-alumina. The samples were heated between 30 and 500 °C, with a heating rate of 10 °C/min, in a nitrogen atmosphere with a flow of 70 mL/min.

#### 2.3.4. Physical–Chemical Characterization of the Scaffolds

The relative density of the matrices was determined by dividing the mass of the sample by its volume, considered cylindrical. Thus, the relative density (*d*) was calculated with the following formula:

(2) $$d=\frac{m}{\pi \;\times \;r^{2}\times \;h}$$

where *d* is the relative density (g/cm^3^); *m* is the sample mass (g); *r* is the radius (cm); *h* is the height (cm).

The relative porosity was determined by measuring the volume of distilled water absorbed by the sample mass in the process. The relative porosity was calculated with the following formula:

(3) $$P=\frac{V_{H_{2}O\;abs}}{m}$$

where *P* is the relative porosity (cm^3^/g); *V_H_2_O__abs_* is the water volume absorbed by sample until equilibrium was reached (cm^3^); *m* is the sample mass (g).

The water retention capacity was determined by immersing the matrix with the initial known mass in distilled water for 48 h, followed by draining and weighing. The water retention capacity (*WRC*) was calculated with the following formula:

(4) $$WRC(\%)=\frac{m_{f}-m_{0}}{m_{0}}\cdot 100$$

where *m_f_* is the final mass of the sample (g); *m*_0_ is the initial mass of the sample (g).

The tensile strength (kN/m^2^) was determined as the pressure applied to break a cylindrical-shaped scaffold (4 cm diameter/1 cm thickness). The parameter was calculated as follows:

(5) $$TS=\frac{2\times m\times g}{\pi \times d\times h}$$

where *TS* is the tensile strength (kN/m^2^); *m* is the mass that breaks the scaffold (kg); *g* is the gravitational acceleration (9.81 m/s^2^); *d* is the thickness of the wire by which the matrix is suspended (m); *h* is the matrix thickness (m);

The wettability of the scaffolds was determined by measuring the contact angle by using the ImageJ 2020 measurement tool. The images in .jpeg format at minimum compression were captured by using a Canon 80D digital single reflex camera (Canon, Tokyo, Japan) and were saved as the camera’s default format, at maximum resolution.

The glucose uptake and release were investigated by immersing a weighted piece of matrix in 10 mL of 0.25% glucose solution for 3 h at 37 °C. The concentration of the glucose not absorbed was established using a BTS-350 spectrophotometer (BioSystems, Barcelona, Spain). Glucose generates, through the coupled Reactions (6) and (7), a colored complex that is spectrophotometrically measured. These reactions are given as follows:

(6) $$glucose+\frac{1}{2}O_{2}+H_{2}O\;\overset{glucosidase}{\to }gluconic\;acid+H_{2}O_{2}\;\;$$

(7) $$H_{2}O_{2}+4-aminoantipirine+phenol\;\overset{peroxidase}{\to }\;quinonimine+4H_{2}O$$

The released glucose was determined by immersing the 3D scaffolds obtained after the glucose uptake in distilled water for 24 h at 37 °C, followed by the determination of the glucose concentration according to the above-described procedure.

The absorption and release of albumin were carried out by immersing a weighted piece of matrix with the known mass in 5 mL of albumin solution (49.2 g/L) prepared in a phosphate buffer solution with pH 7.2. The phosphate buffer solution was prepared by combining 20 mL of monopotassium phosphate solution (KH_2_PO_4_) (9.8 g/L) with 80 mL of disodium phosphate solution (Na_2_HPO_4_) (9.479 g/L). The samples were stored for 3 h at 37 °C and were analyzed using the BioSystems BTS-350 spectrophotometer (Biosystem, Barcelona, Spain). The albumin in the sample reacts with bromocresol green in an acidic medium, forming a colored complex that can be measured spectrophotometrically at 630 nm.

The released albumin was determined by immersing the 3D scaffolds obtained after the albumin uptake in distilled water for 24 h at 37 °C, followed by the determination of the albumin concentration according to the above-described procedure.

#### 2.3.5. Biochemical Characterization of the Scaffolds

In vitro biodegradability under the action of pancreatic lipase was determined by monitoring the mass variation of the matrix after 7, 14, and 21 days of storage in solution containing the enzyme dissolved in sodium phosphate buffer (PBS) solution (50 mM) (pH 7.5) with different concentrations (60 µg/mL, 40 µg/mL, and 20 µg/mL, respectively), following storage at 37 °C. Biodegradability (*B*) was determined with the following formula:

(8) $$B(\%)=\frac{m_{0}-m_{f}}{m_{0}}\cdot 100$$

where *m_f_* is the final mass of the sample (g); *m*_0_ is the initial mass of the sample (g).

The absorption capacity of blood components was monitored by immersing the scaffold in 2 mL of freshly collected pig’s blood on anticoagulant (citric acid). The samples were kept for 24 h at 37 °C, after which they were washed with distilled water. The appearance of the samples after fixation of the blood components was analyzed both macroscopically and using a Bresser optical microscope (10× magnification) (Bresser GmbH, Rhede, Germany). The capacity of the scaffold to absorb the blood components was tested by recording the UV–Vis spectrum (250–700 nm) of the blood remaining after immersing the scaffold and determining the absorbance with the highest units, namely at 278, 347, 412, 544, and 579 nm, respectively. A Perkin Elmer Lambda 35 spectrophotometer (Perkin Elmer, Shelton, CT, USA) was used. The amount of the absorbed blood components by the scaffolds was determined as the measured absorbance (a.u.)/mL absorbed blood/g scaffold. The University Ethics Commission of the Technical University of Cluj Napoca approved the manipulation of biological products for this study (registration no. 667/6.12.2024).

The microbiological biocompatibility of the scaffolds was determined by testing the development capacity of *Lactobacillus plantarum*. The circular-shaped sample (1 cm in diameter) was sterilized by immersion in 75% ethanol solution for 24 h at room temperature, then washed with phosphate buffer solution of pH 7.2 sterilized at 121 °C, for 15 min. The sterilized sample was immersed in MRS agar medium (Oxoid Ltd., Basingstoke, UK) sterilized and cooled at 45 °C and left for 2 h for soaking, after which it was seeded with 0.5 mL suspension containing 5.5 × 10^8^ colonies of *Lactobacillus plantarum*. The samples were analyzed at different time intervals and, in parallel, in vivo microscopic preparations were performed to evaluate the developed *Lactobacillus* culture.

#### 2.3.6. Statistical Analysis

Density, porosity, WRC, tensile strength, and glucose, albumin, and blood components uptake were investigated in triplicate and the mean and standard deviation were determined according to the mathematical procedure described by Mihaly Cozmuta [15]. Moreover, the statistical significance was determined in Microsoft Excel, using one-way ANOVA processing and the Tukey model.

## 3. Results and Discussion

### 3.1. Morphology and Elemental Analysis

Macroscopically, the samples are white. Those prepared by using a higher amount of sodium chloride (M2, M4, and M6) are more porous and softer when handled (Table 1), while those prepared using less sodium chloride (M1, M3, and M5) are more compact and a little rougher when handled (Table 1). There is no macroscopically observable difference between PLA and PCL.

The optical microscopy images show that the PLA filaments in the M1 and M2 samples as well as the PCL filaments in the M3 and M4 samples are interconnected (Table 2). Differences between the PLA samples (M1 and M2) and the PCL samples (M3 and M4) appear, namely that the edge of the PLA matrix is more rounded and does not form filamentous ends, explained by the shorter chain, in comparison with the long linear hydrocarbonate chain of the PCL structure. This also explains the more rigid aspect of PLA and the more flexible characteristics of PCL [10,11]. In contrast, the microscopic images of the M3 and M4 samples show a more filamentous structure given by a larger number of methylene groups from the PCL structure (Table 2). The M3 sample is more compact than the M4 sample. A combination of compact and lax phases, corresponding to PLA and PCL, respectively, is observed in the case of PLA-PCL matrices (M5 and M6 samples).

The SEM images (Table 2) show a compact structure in which the pores generated because of the porogen dissolution can be seen. The samples prepared with the higher mass of porogen are, as expected, more porous, containing larger pores than those of the samples with the lower amount of porogen. The surfaces of the M1 and M2 samples (PLA samples) are rougher than those of the M3 and M4 (PCL) and M5 and M6 (PLA-PCL) samples. The addition of PCL improves the overall toughness of the PLA-PCL matrix, a result confirmed by those of Eyiridiz et al. [10] and Solechan et al. [11]. The SEM image of the M3 sample also shows the filamentous areas (blue circles) confirmed by the optical images. Hussain et al. [16] also developed PLA-based films modified with graphene oxide and their SEM images showed compact, homogenous, and smooth surfaces, indicating the high quality of the PLA films. They also proved, via SEM images, the presence of reinforcements in the PLA matrix. Zain et al. [17] also prepared PLA-PCL scaffold samples that exhibited a wide pore size distribution (ranging from 100 to 500 μm).

Table 3 illustrates the EDXS results. Carbon ranges between 55.6 and 72.5 wt%, while oxygen ranges between 27.5 and 44.4 wt%. The presence of chloride, sodium, and kalium in M1, M2, and M6 samples revealed that the porogen was not completely removed during washing, but the remaining amounts are very low. This suggest that the washing is an important step in materials development. The amount of Na, K, and Cl is very small in this case and, as will be seen later, it does not significantly influence the biocompatibility and the degree of biodegradability of the materials.

### 3.2. Structure of the Scaffolds

**Figure 1:**
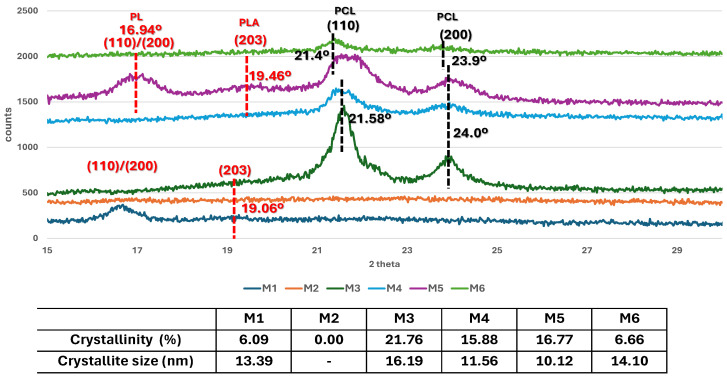
XRD patterns, crystallinity, and crystallite size of the scaffolds.

The XRD profiles of the scaffolds are presented in Figure 1. Generally, PLA crystallizes in α, α′, β, and γ crystal forms depending on the different crystallization conditions [18].

The PLA-containing matrices prepared with the lowest amount of porogen (M1 and M5) show an intense crystallization reflection at 16.64° in the M1 sample, suggesting the presence of PLA in the α crystalline form [19,20], results confirmed by the thermogravimetric analyses. DTA showed that the melting point for PLA was in the range 152–154 °C, characteristic for α crystalline PLA [21]. This signal was shifted to 16.94° in the M5 sample, indicating that the presence of PCL slightly decreased the PLA’s crystallinity. This reflection does not appear in the XRD profiles of the samples prepared using the highest amount of porogen (M2 and M6). A sharp reflection appears at 19.06° and 19.46°, respectively, in the M1 and M5 samples, corresponding to the (110)/(200) plane of the PLA crystal structure [21].

Two strong reflections at 21.58° and 24°, corresponding to the (110) and (200) planes of the orthorhombic crystal structure of PCL, respectively, appear in the XRD profiles of the M3 and M4 samples [1,22]. These reflections are shifted to 21.4° and 23.9°, respectively, in M5 (the PLA-PCL composite with the lowest porosity) as a result of PLA influence, and are considerably reduced in M6 (the PLA-PCL composite with the highest porosity).

The highest crystallinity was determined in the PCL-based scaffolds (samples M3 and M4), followed by the mixed materials (M5 and M6) and the lowest was determined in the PLA-based ones (samples M1 and M2). The better crystallinity is prevailingly due to the minimized formation of defects [3]. It can be noted that in both PLA- and PCL-based samples, the degree of crystallization decreases with the amount of porogen and, implicitly, with the porosity. An explanation could be as follows: crystallization involves two stages, namely nucleation and crystal growth; the crystallization centers are formed by the aggregation of particles during the nucleation process; subsequently, new particles are attached to the formed aggregates (crystallization centers) and, thus, crystal growth takes place. Nucleation is favored by the small distance between particles and molecules [18]. The carbonyl groups in the polymer structure generate inter- and intramolecular interactions, such as hydrogen bonds, which bring the polymeric chains closer together, favoring nucleation. The functional groups are spaced in the porous samples, the number of hydrogen bonds decreases and, implicitly, the polymer chains are distanced, thus reducing nucleation and, finally, crystallization [18]. The highest crystallinity degree and the largest crystallites in size have been determined for the PCL-based samples (M3 and M4), with this approach contributing to the superior mechanical properties demonstrated by tensile testing. The results agree with those of Kumar et al. [3] who developed PCL-based filaments with different arrangements, suggesting that a high crystallinity degree of the materials confer a good mechanical resistance. The lowest structural characteristics correspond to the PLA-based samples (M1 and M2). Moreover, according to Abdelhameed et al. [22], a reduced crystallinity degree as a result of the porosity can be also explained by the more predominant defects generated in the composite structure in comparison with the individual material (neat PCL). These defects could appear as a result of the more distanced groups and chains.

However, the mixed PLA-PCL samples (M5 and M6) contain reflections characteristic of both PLA and PCL. PCL filaments are well dispersed into PLA ones, forming bonds because they have good adhesive properties [11]. Furthermore, the addition of PCL lowered the percentage of crystallinity in the PLA-PCL matrix compared with pure PCL, which was caused by PLA crystallization interfering with PCL crystallization [11]. Soltani Gerdefaramarzi et al. [8] prepared graphene-modified PLA-PCL matrices and made the same observation. They noted that the mixed matrix contained signals specific to both types of polymers. The degree of crystallization and the size of the crystallites of mixed PLA-PCL matrices are between those of individual PLA and PCL matrices. As regards the less porous samples, the crystallinity and the size of the crystallites of the PLA-PCL mixed sample (M5) are bigger than those of the PLA (M1 sample) and smaller than those of the PCL (M3 sample). The same variation is observed in the porous samples. In this regard, PCL and PLA-PCL scaffolds are more suitable for the manufacturing of smart scaffolds than PLA ones, because they present a higher crystallinity degree.

**Figure 2:**
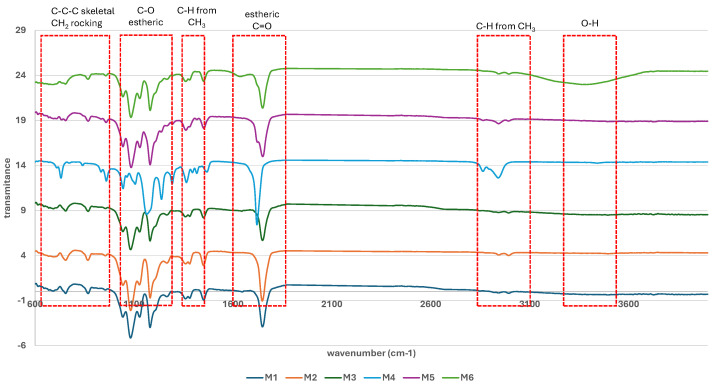
Standardized FTIR spectra of the scaffolds.

The chart in Figure 2 shows the standardized FTIR spectra of the samples. The standardized values of transmittance were calculated with the following formula:

Standardized value = (experimental value − average)/standard deviation(9)

Characteristic vibrations for the O–H stretching at 3395 cm^−1^ are more pronounced in the FTIR spectrum of the M6 sample [8,17]. Since PLA and PCL are esters and do not contain a significant amount of free –OH groups, this band may instead indicate residual moisture (absorbed water), which is common in FTIR spectra of porous samples. This result is positively correlated with water absorption capacity and porosity, which are the highest in the case of the M6 sample (Figures 5 and 6). The signals at 2996 and 2946 cm^−1^ are associated with the asymmetric and symmetric stretching of C–H bonds from CH_3_ groups in both PLA and PCL structures [8,17]. The interval from 1722–1749 cm^−1^ corresponds to the stretching of C=O bonds from the ester groups [23]. In this interval, a signal at 1725 cm^−1^ appears in the FTIR spectrum of the M4 sample, assigned to the C=O stretching from the ester bond in a more amorphous material [24]. Signals at 1750–1752 cm^−1^ which appeared in the FTIR spectra of the other samples are assigned to the vibration of C=O ester bonds from the PLA-PCL mixture [8,25]. These results are in agreement with those achieved from the XRD patterns. Signals in the range 1456–1366 cm^−1^ correspond to the asymmetric deformation of methyl groups in PLA [23] and the scissoring of methylene groups in PCL [26,27,28]. The frequency of 1296 cm^−1^ is present in the FTIR spectra of both PLA- and PCL-based samples and corresponds to the etheric C–O–C stretching and C–H wagging in PLA [23] and C–C stretching and methylene twisting in PCL [24]. The signals in the range 1242–1044 cm^−1^ are attributed to the ester C–O stretching in PLA and ester C–C–O stretching in PCL [24]. The frequencies between 963 and 733 cm^−1^ are relatively specific, and the signals at this interval are assigned to the skeletal C–C, C–H wagging in PLA [29], and to the methylene rocking and C–C–C bending in PCL. As we already noted, the bands from the standardized FTIR spectra of M2, M4, and M6 are slightly misplaced, with the most pronounced displacement being noted in the case of M4. The displacements can be generated by several factors, including crystallinity degree [25] and intermolecular interactions, such as hydrogen bonds [26]. We have discussed above that the M2, M4, and M6 samples contain a more dispersed network as a result of containing the highest amounts of the used porogen. Thus, the inter- and intramolecular interaction preponderance in these samples is lower in comparison with that in samples M1, M3, and M5, and the difference in their density could be the explanation for the bands’ displacement [30].

### 3.3. Differential Thermal Analysis (DTA) and Thermogravimetry (TGA)

**Figure 3:**
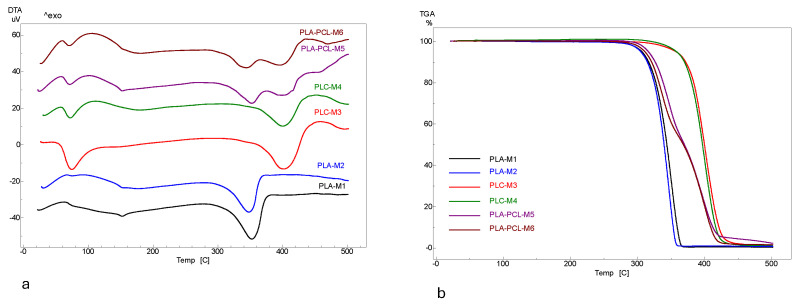
DTA (**a**) and TGA (**b**) profiles of scaffolds.

The thermal behaviour of the scaffolds is observed in the DTA and TGA curves, as illustrated in Figure 3a,b. The maximum temperatures at which DTA events occur, and the mass losses observed in the thermogravimetric curves during the heating process are displayed in Table 4.

Because of their disordered structures, samples M1 and M2 try to reach a more ordered phase when heated at 100 °C. The weak exothermic signals that appears in the DTA curve (Figure 3a) of the two samples at ~63 and ~77 °C, respectively, represent the glass transition temperatures (T_g_), namely the point at which the PLA changes from a rigid, glass-like form to a more flexible, rubbery one [30]. The endothermic signal appearing at 152 °C in sample M1 and at 154 °C in sample M2 is due to the polymer melting (Figure 3 and Table 4). The next endothermic signal with a maximum at ~353 °C in M1 and a maximum at ~ 347 °C in M2 is associated with the thermal degradation of PLA, via cleavage of the ester bonds and the release of CO_2_, lactic acids and other volatile products [13]. Thermogravimetric data showed (Figure 3b) that samples M1 and M2 have thermal stability up to 273 °C and 275 °C, respectively. The weight loss due to polymer decomposition was at 315 °C ~8% in M1 and ~8.9% in M2, respectively.

The first endothermic signal with a maximum of 75 °C for M3, and 72 °C for M4, occurs because of the melting of the PCL polymer. The broad endothermic signal with the maximum at 181 °C in the DTA curve of sample M4 could indicate crystallization, recrystallization, or relaxation. A difference between M3 and M4 appears in the range of 310–380 °C, namely the mass loss for M3 was 8.6% and for M4 was greater (19.1%). This shows that the decomposition begins earlier in the case of M4 sample, probably as a result of higher porosity. The porous sample is less compact, and the ending groups are more thermally vulnerable [10,31]. The endothermic events at the maximum peaks of ~402 °C in M3 and at ~400 °C in M4 are due to the thermal degradation of the polymer, through pyrolysis and ester bond cleavage processes [31]. The exothermic signals located at 455 and 453 °C can be associated with the complete combustion of degradation residues. The thermogravimetric curve (Figure 3b) shows the thermal stability of samples M3 and M4 up to 310 °C, after which polymer decomposition starts. The weight loss at ~370 °C due to decomposition is ~8.6% in M3 and ~10.2% in M4, respectively.

In the samples prepared from mixed PLA and PCL polymers (M5 and M6), the melting of PCL polymer is associated with endothermic signals from 72 °C in M5 and at 71 °C in M6. The melting of the second PLA polymer is observed in the presence of the endothermic signal observed at 153 °C in M5 and 154 °C in M6. The next two endothermic signals appear due to the PLA and PCL polymer chain decomposition and are observed at 352 and 398 °C in the DTA curve of M5, respectively, and at 344 and 396 °C in the DTA curve of the M6 sample, respectively. The exothermic signal due to the combustion of PCL residues appears at a lower value in the combined samples, i.e., at 430 °C in M5 and at 436 °C in M6.

Comparative analysis of DTA curves shows that melting and decomposition occur at different temperatures in the mixed samples (PLA-PCL) compared to in the individual samples (PLA and PCL, respectively). The TGA curves show a delayed decomposition in the M3 and M4 samples compared to the M1 and M2 samples. By following the shape of the TG curves, it can be observed that the mass losses in the mixed PLA-PCL samples occur in two stages. The presence of PLA in the PCL matrix generates a slightly less stable composite than neat PCL. Gurler et al. [32], who developed PCL-based materials modified with a photopolymer designed for food packaging, showed a decrease in the thermal stability of PCL as the amount of photopolymer increased by reducing the glass transition temperature (T_g_) of PCL. The TGA-DTA results are correlated with the XRD results, namely the more stable materials are PCL-based materials displaying the highest crystallization degree. Kumar et al. [3] showed that crystallinity is one of the most important thermal property for scaffold manufacturing and that the more thermally stable materials are those which are better crystallized. By corroborating the results with the data in the literature, it can be concluded that porosity is not significant from the thermal stability point of view and both PCL-based scaffolds (the less porous M3 and the more porous M4) are better candidates for scaffold manufacturing in tissue engineering than PLA-based or PLA-PCL materials. Eryildiz et al. [10] showed that T_g_ values for the PLA-PCL blends are slightly lower than pure PLA due to the influence of semi-crystalline PCL molecular chains on the PLA crystallization and to the plasticizing effect of PCL, which is a more flexible and ductile polymer compared to the more rigid PLA [10].

### 3.4. Physical–Chemical Characterization of the Scaffolds

**Figure 4:**
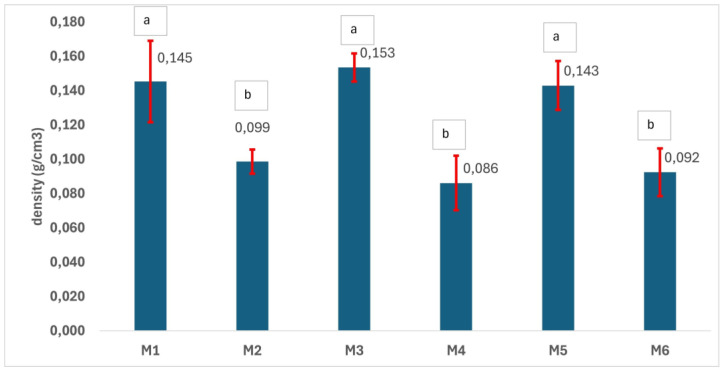
Density of the 3D scaffolds (a, b—the same letter refers to nonsignificant differences, based on the one-way ANOVA/Tukey model).

**Figure 5:**
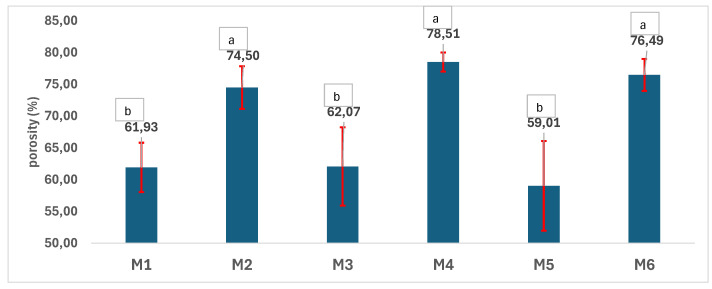
Porosity of the 3D scaffolds (a, b—the same letter refers to nonsignificant differences, based on the one-way ANOVA/Tukey model).

**Figure 6:**
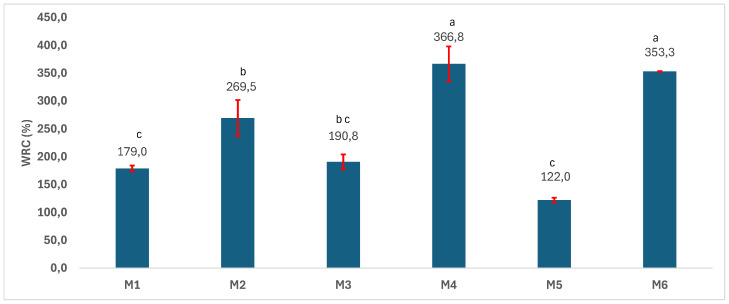
Water retention capacity (WRC) of the 3D scaffolds (a, b, c—the same letter refers to non-significant differences, based on the one-way ANOVA/Tukey model).

**Figure 7:**
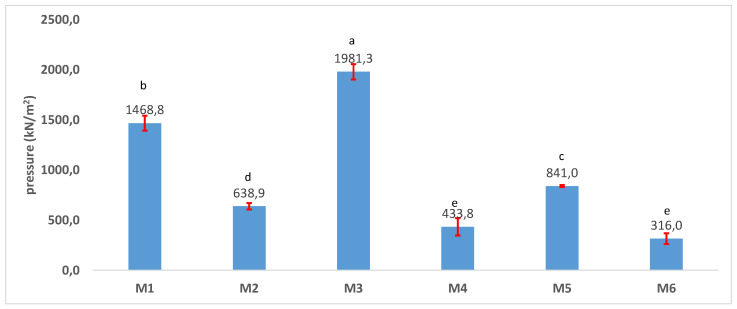
Tensile resistance of the 3D scaffolds (a, b, c, d, e—the same letter refers to nonsignificant differences, based on the one-way ANOVA/Tukey model).

**Figure 8:**
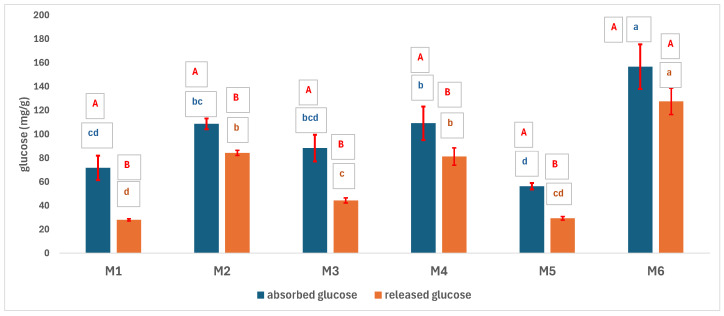
Absorbed and released glucose from the 3D scaffolds (the capital letters show whether the difference between absorbed and released glucose is statistically significant in the case of the same type of matrix, based on the one-way ANOVA/Tukey model; if it is the same letter, the difference is not statistically significant. The small blue letters show the difference in statistical significance between the matrices in the case of absorbed glucose, based on the one-way ANOVA/Tukey model; the same letter represents a statistically insignificant difference. The small orange letters show the difference in statistical significance between the matrices in the case of the glucose released, based on the one-way ANOVA/Tukey model; the same letter indicates a statistically insignificant difference).

**Figure 9:**
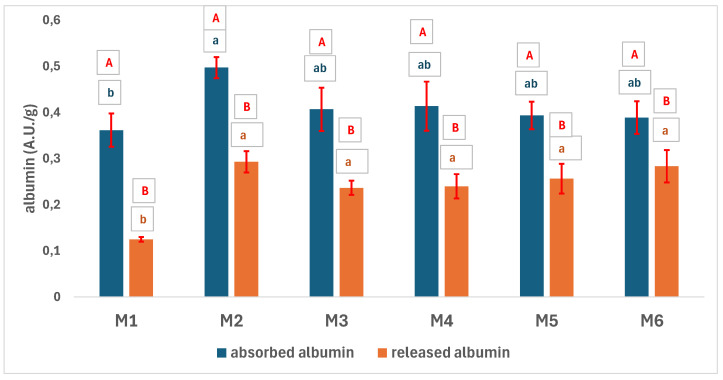
Absorbed and released albumin from the 3D scaffolds (the capital letters show whether the difference between absorbed and released glucose is statistically significant in the case of the same type of matrix, based on the one-way ANOVA/Tukey model; if it is the same letter, the difference is not statistically significant. The small blue letters show the difference in statistical significance between the matrices in the case of absorbed glucose, based on the one-way ANOVA/Tukey model; the same letter indicates a statistically insignificant difference. The small orange letters show the difference in statistical significance between the matrices in the case of the glucose released, based on the one-way ANOVA/Tukey model; the same letter represents a statistically insignificant difference).

The charts in Figure 4, Figure 5, Figure 6, Figure 7, Figure 8 and Figure 9 reveal the physical–chemical characteristics of the scaffolds. Samples M1, M3, and M5 are denser than samples M2, M4, and M6, which were prepared using a double amount of NaCl porogen (Figure 4). No statistical difference between samples M1, M3, and M5, as well as between samples M2, M4, and M6, in terms of density can be noted. In contrast, the difference between the density values as a function of the amount of porogen used is statistically significant according to the one-way ANOVA/Tukey model (*p* > 0.05) (Figure 4). As expected, the porosity of the scaffolds shows an inversely proportional variation with density. The most porous samples are M2, M4, and M6, with porosity values between 74% and 78.5%, while the porosity values of M1, M3, and M5 are in the 59–62% range (Figure 5). No significant difference in terms of porosity could be determined between M1, M3, and M5 as well as between M2, M4, and M6, meaning that the porosity is not influenced by the polymer type or by the mixing of the two materials. Soltani Gerdefaramarzi et al. [8] also determined the porosity of PLA and PCL scaffolds modified with graphene as being between 72% and 77%, similar to that of the porous samples M2, M4, and M6 developed in this study. Additionally, they obtained similar porosity both for the PLA and the PCL samples.

Hydrophilicity is an important parameter of the scaffolds used as materials in tissue reconstruction. The topography and hydrophilicity of the surface of scaffolds are the key factors in nutrient molecules and in terms of cell adhesion. The water droplets on the 3D scaffold’s surface and the average contact angles are displayed in Table 5, according to which all samples are hydrophilic. The differences between the contact angles are statistically nonsignificant. Hydrophilic materials are those that form a contact angle of less than 90° [33]. Theoretically, PLA is hydrophilic [34], while PCL is hydrophobic [35]. In our case, contact angles below 90° were also obtained for PCL-based scaffolds, meaning that the materials are hydrophilic. This behavior, in opposition to the literature, could be explained by the morphology of the surface on which the drop falls. The contact angle formed by the droplet falling on the porous area is smaller than the angle formed on the pore-free area. Therefore, the high porosity of the PCL-based scaffolds is responsible for their hydrophilic character. Contact angle measurements on highly porous surfaces can yield lower angles (greater “apparent” hydrophilicity), due to the Wenzel or Cassie–Baxter effects [25]. The literature data revealed that the microporous structure could also be responsible for improving the hydrophilicity of a material [27].

Overall, these results show that PLA, PCL, and PLA-PCL scaffolds seem to be conductive to nutrients and cell adhesion, and as a matrix for any kind of tissue engineering requiring a porous structure and good wettability [36].

The water retention capacity (WRC) reflects the scaffolds’ ability to absorb body fluids, facilitate the transfer of essential nutrients and metabolites, and allow cell adhesion, proliferation, and differentiation. As expected, the water retention capacity (Figure 6) is significantly higher (one-way ANOVA/Tukey model (*p* > 0.05)) for the most porous samples (M2, M4, and M6) as compared to M1, M3, and M5, because of their higher porosity. The porous samples based on PCL (M4) and PCL-PLA (M6) have a higher WRC as compared to the neat PLA (M2). Theoretically, WRC would be correlated with the presence of the hydrophilic groups as well as with the microporous structure of the matrix. In our case, the share of OH and ester groups is higher in the M2 matrix (PLA) than in M4 matrix (PCL). Moreover, M2 and M4 have similar porosity (no significant difference between them in terms of porosity could be noted, according to the one-way ANOVA/Tukey model). Even so, the WRC of the PLA (M2) sample is significantly smaller than that of the PCL (M4) and PLA-PCL, respectively (M6), although, they should be similar, based on the above-presented characteristics. In this regard, M2’s behavior could be explained by the different pore size, as M2 contains small pores where water molecules cannot penetrate, thus leading to a lower WRC. Soltani Gerdefaramarzi et al. [8] developed PLA scaffolds with a pore size smaller than that of PLA-PCL and PCL, respectively. The conclusion is also similar to the statement of Garcia et al. [27], who developed PCL based-scaffolds modified with starch and/or CaO. In their case, the water absorption capacity increased with the content of starch and CaO, but, in addition, the authors also suggested that the microporous structure of the composites could also enhance the water absorption capacity.

PCL is stronger mechanically than PLA and PLA-PCL composites, due to a more crystallized structure [8,10,11], as revealed by the results for mechanical strength presented in Figure 7. The results for mechanical strength are correlated with the conclusions of the XRD and FTIR measurements because of a smaller number of hydrogen bonds and a lower crystallinity conferring a smaller mechanical strength to the material in the porous samples. The results agree with those of Senatov et al. [37] who showed that the presence of PCL, because of its high elasticity, considerably increased the elastic modulus of PCL-PLA. Our study also proved that the mechanical resistance of PCL is significantly higher than that of PLA (*p* > 0.05 according to the one-way ANOVA/Tukey model). The PCL-based samples are more crystallized as compared to PLA. More crystallization contributes to better mechanical resistance [3]. The porosity is a factor that also affects the mechanical resistance. The more porous samples were less mechanically resistant. This can be explained by the distancing between the functional groups in the porous samples, thus decreasing the preponderance of the intra- and intermolecular interactions, like hydrogen bonds, and, implicitly, the space between the polymer chains. Therefore, the overall effect is a lower tensile strength. With reduced crystallinity and predominant defects in the composite matrix, the PLA-PCL samples have a smaller tensile resistance as compared to PCL and PLA [22]. Eryildiz et al. [10] concluded that PCL provides greater flexibility and toughness to the PLA-PCL blend, because PLA is highly brittle and PCL is highly ductile. Thus, PCL functions as a toughening agent for the PLA matrix.

M6, the porous PLA-PCL sample, uptakes more glucose than the other samples (Figure 8). The difference between M1, M2, M3, and M4 is not statistically significant. The absorption capacity of M1, M3, and M5, i.e., the less porous samples, is the smallest. The absorption of glucose on PLA is intense due to the compatibility between the OH groups of the carbohydrate and those of the PLA and due to its hydrophilic property, while the most intense absorption by M6 occurs because of the four-factor synergism of porosity, crystallinity, polar compatibility, and pore size. The results are in tune with those obtained at the WRC tests and are similar to those in the literature [8,27]. Additionally, Green et al. [38] showed that glyco-PLA copolymers exhibited a successful uptake of both hydrophobic and hydrophilic molecules. All the samples released an amount of glucose smaller than the absorbed one, which is valuable result for the aim of the study, namely the use of the developed matrix for tissue reconstruction. The explanation could be the physical adsorption of glucose into the matrix and the chemical bonds generated (eteric and estheric) between the OH groups of glucose and the marginal carboxyl groups of the polymer. The physical bonds are broken during the release in more intense manner than the chemical ones, thus explaining the difference between uptake and release. The trend of all samples is similar to the trend spotted for the uptake profiles. The amount of glucose released increases with porosity and is considerably higher in the case of mixed PLA-PCL scaffolds. No statistical differences between the PLA and PCL samples can be noted.

All the samples have a similar uptake of albumin (Figure 9). M1 differs significantly from M2 (*p* > 0.05 according to the one-way ANOVA/Tukey model), as M1 has a smaller absorption capacity than the other samples because of its low porosity and the high volume of the albumin molecule. The amount of albumin absorbed statistically exceeds the amount of albumin released. The albumin released and, respectively, absorbed has a similar trend of variation. There is no significant difference depending on the type and porosity of the samples in terms of albumin released.

Senatov et al. [37] proved the efficient uptake and release capacity of proteins given by the scaffolds based on PLA-PCL–hyaluronic acid. They stated that the different release kinetics of the materials indicate that the matrix itself makes a definite, and quite large, contribution to protein retention.

### 3.5. Biochemical Characterization of the Scaffolds

#### 3.5.1. In Vitro Biodegradability

**Figure 10:**
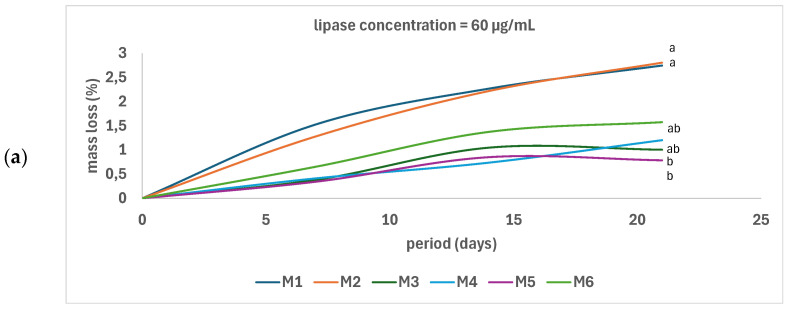

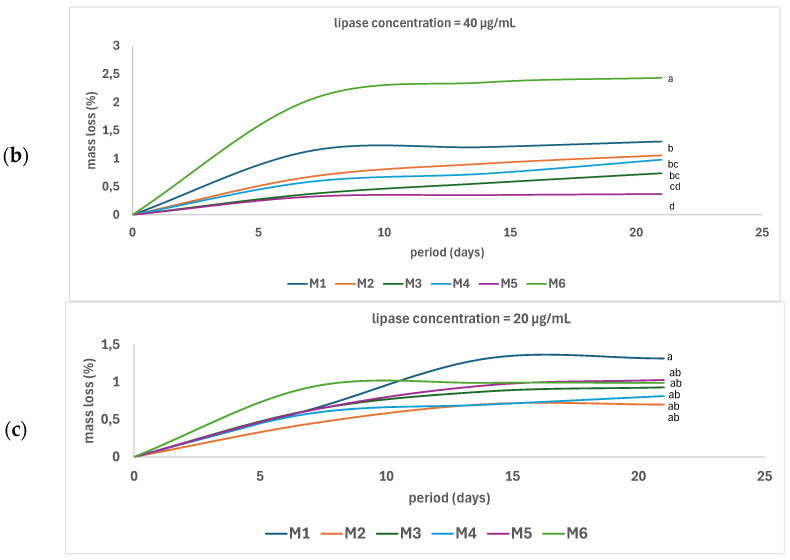
In vitro biodegradability of the scaffolds in lipase solutions with concentration of 60 µg/mL (**a**), 40 µg/mL (**b**), and 20 µg/mL (**c**) at 37 °C (the same letters represent that the differences are statistically nonsignificant according to one-way ANOVA/Tukey model).

In vitro biodegradability was determined by monitoring the mass decrease in matrices immersed in lipase solutions of different concentrations. The mass loss increased with the enzyme concentration (Figure 10). At a 20 μg/mL concentration of lipase, there was no significant difference between samples regardless of type and porosity. At a lipase concentration of 40 μg/mL, the greatest mass loss was determined in the case of the M6 sample (porous PLA-PCL), followed by the PLA-based samples, and the most stable were the PCL-based samples. At an enzyme concentration of 60 μg/mL, in a similar trend, the PLA and PLA-PCL samples are more vulnerable to lipase, while the PCL samples are the most stable. Theoretically, PLA is not highly susceptible to degradation by pure lipases. Lipases generally act more effectively on PCL and PHB [39]. The biodegradability of PLA, which is higher than that of PCL, is explained by the fact that the lipase solution was prepared in aqueous sodium phosphate-buffered media (PBS). This behavior is due to the prior cleavage of ester bonds under the catalysis of lipase enzymes and the preponderance of ester bonds in PLA, which is higher than in PCL [39]. Moreover, the thermodynamic stability of the hydrocarbon linear chain (–(CH_2_)_5_–) in PCL is higher than the thermodynamic stability of the (–CH(CH_3_)–) group in PLA [39]. Another factor affecting the enzymatic degradation is crystallinity. The presence of the crystalline zones prevents the diffusion and distribution of the enzyme within the sample, and, thus, the biodegradability of the matrix. In our study, the less crystallized samples are M1 and M2 (PLA-based samples) and M6 (porous PLA-PCL), displaying the most intense enzymatic degradation. In this regard, PLA and PLA-PCL materials could be used to develop self-degradable composites with controlled lifetimes. Although, generally, PCL is more degradable than PLA, in this case the stability of PCL scaffolds exceeds the stability of PLA because of the action of lipase, as PCL scaffolds have a cleaner-cut semicrystalline structure [27]. Because of this, a PCL scaffold is a valuable option for reconstructing digestive tissues. Hussain et al. [16] who tested the biodegradability of the PLA-based samples modified with graphene oxide (GO) and graphene nanoparticles (GNPs), respectively, also showed the intense degradability of PLA in phosphate-buffered saline and Ringer’s solutions, but PLA/GO, PLA/GNP, and PLA/CNTs composites exhibited slow degradation as compared to pure PLA. Jamali et al. [28], who developed hydrogels based on chitosan modified with PCL fibers, also showed that the biodegradability (weight loss) arises from chitosan and not PCL, because PCL loses its weight over a long period of time. These results are also supported by Garcia and coworkers [27], who proved the low biodegradability of neat PCL in comparison with the composites obtained by modifying PCL with starch and/or CaO. The results were similar also to those obtained by Soltani Gerdefaramarzi et al. [8] and Zain et al. [17], meaning that the degradation rate of PLA-PCL composite is higher than that of PCL.

#### 3.5.2. The Absorption of Blood Components on the Scaffolds

One of the most important factors in manufacturing scaffolds is cell adhesion. The images in Table 6 show that the most porous samples (M2, M4, and M6) have their entire surfaces covered with blood, in comparison with samples M1, M3, and M5, which show only some red areas. The section images also show that samples M2, M4, and M6 are better impregnated with blood than samples M1, M3, and M5, suggesting that the absorption capacity is positively correlated with porosity, hydrophilicity, and roughness. The microscopic images support the results given by the macroscopic images.

**Figure 11:**
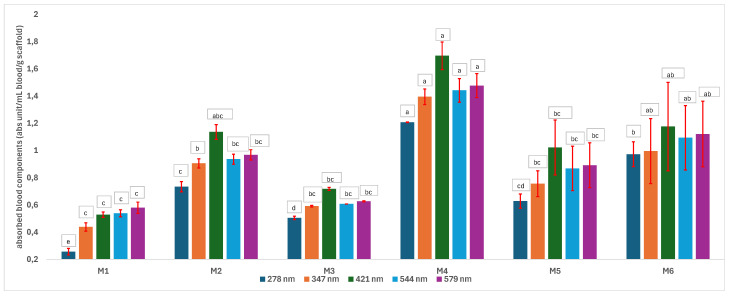
Levels of absorbed blood components onto scaffolds (a, b, c, d, e—the same lowercase letter refers to nonsignificant differences, based on the one-way ANOVA/Tukey model).

The analysis of data in Figure 11 reveals the same results. The highest values for the absorbed blood components were determined for M2, M4, and M6, which are the most porous, hydrophilic, and present the most reduced roughness. No statistical significance was recorded between these three samples, and no difference between M1, M3, and M5 could be observed. When analyzing Figure 11, it is observed that the absorption capacity of the M2 sample is lower than that of M4 and M6, although the porosity is high (Figure 5). This suggest that another important factor affecting absorption is hydrophilicity. M2 is less hydrophilic than M4 and M6. In addition, the reduced absorption capacity could be also because the M2 sample, although porous, contains smaller pores that do not allow the penetration of blood cells and molecules into the plasma composition. This result confirms the conclusion regarding the WRC, with M2 presenting the most reduced absorption in comparison with the other porous samples.

Peiravi et al. [40] developed layer-by-layer PCL/PLA samples with more hydrophilic surfaces than pure PCL samples, which can improve the adhesion, growth and proliferation and differentiation to bone cells, and intensified calcium mineralization.

#### 3.5.3. The Microbiological Biocompatibility of the Scaffolds

The microbiological biocompatibility of the matrices was determined by testing the development capacity of the *Lactobacillus* strains sown on the matrix soaked in the appropriate nutritive medium. The purpose of this analysis was to establish whether lactobacilli strains develop on the tested matrices, thus giving valuable information about their probiotic support capacity. On the surface of all matrices covered with the nutritive medium, *Lactobacillus* colonies developed to a smaller extent after two days and were more pronounced after five days, with the aspect of a whitish biofilm. The analysis of these materials, as shown in Table 7, reveals that this phenomenon is more prominent in the porous (M2, M4, and M6) samples and the PLA-PCL samples. Peng et al. [41] showed that the PCL microplastic particles exhibited stability in the upper gastrointestinal tract, while PLA microplastic particles were degraded beginning in the small intestine digestion phase. They also proved that microparticles of PCL and PLA exhibited the same inhibitory effects on key probiotics, such as *Bifidobacterium*, *Lactobacillus*, *Faecalibacterium*, *Limosilactobacillus*, *Blautia*, *Romboutsia*, and *Ruminococcus*, which highlighted the potential hazards of these materials for human health. In our study, large matrices were used that were covered with nutrient media, which facilitated the development of lactobacillus strains. Thus, the non-toxic character of the matrices based on both PLA and PCL is highlighted. The same non-toxic character of the PLA and/or PCL was also shown by Soltani Gerdefaramarzi et al. [8], who demonstrated the non-toxicity of PLA, PCL, and PLA-PCL scaffolds for the PC-12 cells (a cell line derived from a pheochromocytoma of the rat adrenal medulla). Their results showed a good fixation capacity and support for the efficient development of porous samples for living cells.

## 4. Conclusions

The study aimed to develop materials based on polylactic acid (PLA) and/or polycaprolactone (PCL) by porogen leaching as potential scaffolds for tissue reconstruction and to establish the influence of porosity on scaffold characteristics. PCL and PCL-PLA matrices are more suitable options for reconstructing tissues than neat PLA, because, based on their higher degree of crystallization, they are more stable and stiffer. Porosity reduced the degree of crystallization, the size of crystals and the preponderance of hydrogen bonds. The mixed PLA-PCL samples have a lower degree of crystallization than the PCL samples. The FTIR analyses confirm the XRD results, in terms of the lower share of intra- and intermolecular interactions in the more porous samples compared to the less porous samples, which is demonstrated by the displacement of the peaks in the FTIR spectra of the more porous samples. The PCL samples are thermally the most stable. Mixed PLA-PCL samples thermally decompose into two temperature stages and are less stable than PCL. The porosity, the degree of crystallization, the share of hydrophilic groups, and the pore size influence the absorption capacity of water, glucose, albumin, and blood components, which have both physical and chemical natures. Even though the porosity is high and the sample is amorphous, the reduced water absorption capacity is due to the small size of the pores and a slightly reduced hydrophilicity. PCL is more mechanically resistant than PLA and PLA-PCL. The mechanical resistance decreases as porosity and the degree of crystallization decreases. The high porosity (>78%) and the large pore size induced the “apparent” PCL hydrophilicity due to the Wenzel or Cassie–Baxter effects. PLA and PLA-PCL are more vulnerable to the action of lipase than PCL due to the higher share of ester groups and the lower degree of crystallization. *Lactobacillus* grows as porosity increases and is more accentuated on PLA-PCL, revealing its lack of toxicity, although the absence of toxicity toward a probiotic bacterium does not automatically imply full biocompatibility for human cells. All these results indicate that mixed PLA-PCL matrices are effective candidates for tissue reconstruction. Future studies will consist of additional biological property assessments to further support potential biomedical applications.

## Figures and Tables

**Table 1:** Raw materials and quantities used to prepare the scaffolds.

Sample Code	PLA (g)	PCL (g)	CH_2_Cl_2_ (mL)	Polymer–NaCl (wt:wt)	NaCl (g)	Image
M1	9	-	51	1:8	72	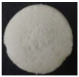
M2	9	-	51	1:16	144	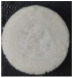
M3	-	9	51	1:8	72	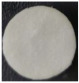
M4	-	9	51	1:16	144	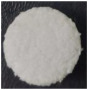
M5	4.5	4.5	51	1:8	72	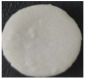
M6	4.5	4.5	51	1:16	144	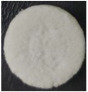
**Washing status of the scaffolds**						
After the first day of washing	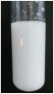	Incomplete washing	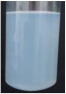	Complete washing		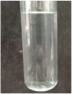

**Table 2:** Microscopic and SEM images of 3D scaffolds.

Sample Code	Microscopic Image (Mag 10×)	SEM Image
M1	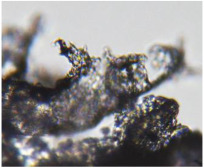	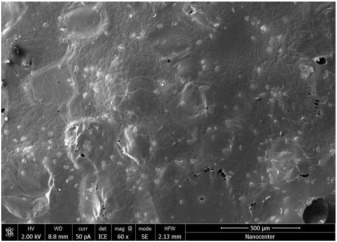
M2	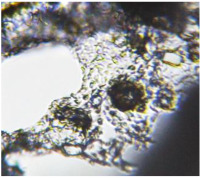	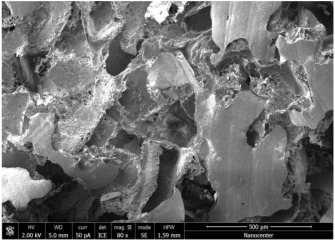
M3	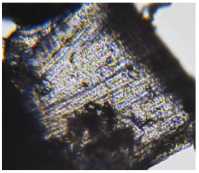	** 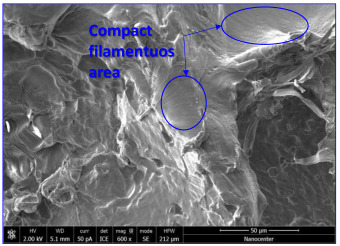 **
M4	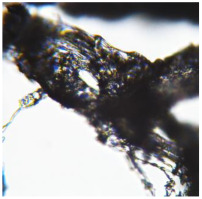	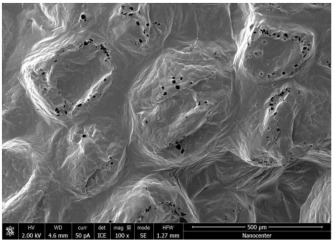
M5	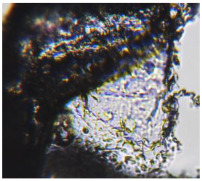	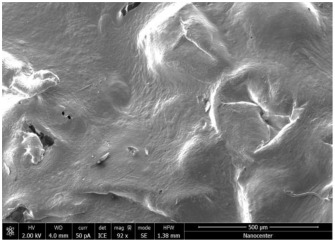
M6	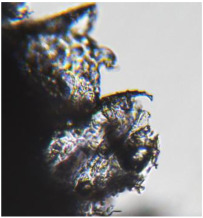	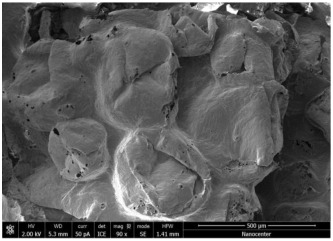

**Table 3:** Map sum spectrum and elemental composition of the matrices resulting from EDXS.

Sample Code	Map Sum Spectrum
M1	** 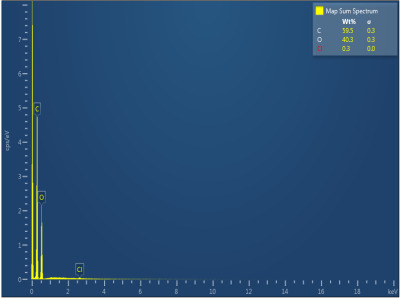 **
M2	** 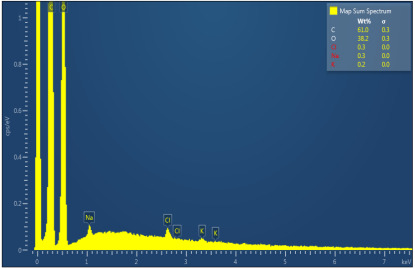 **
M3	** 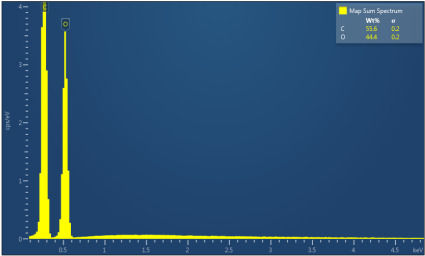 **
M4	** 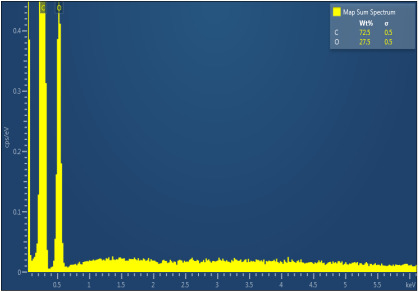 **
M5	** 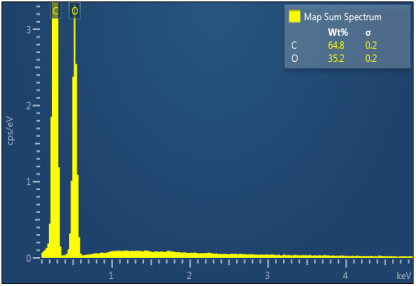 **
M6	** 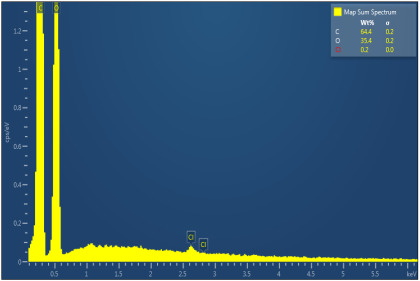 **

**Table 4:** DTA/TGA parameters of the scaffolds.

Sample	DTA Events	T_g_: Mass Loss	Total Mass Loss
M1	Exo 63 °C glass transition	-	−99.0%
	Endo 152 °C melting	-	
	Endo 353 °C decomposition	273–315 °C: −8.0%	
		315–380 °C: −91%	
M2	Exo 77 °C glass transition	-	−98.2%
	Endo 154 °C melting	-	
	Endo 347 °C decomposition	275–315 °C: −8.9%	
		315–380 °C: −89.3%	
M3	Endo 75 °C melting of PCL	-	−99.2%
	Endo 402 °C decomposition	310–370 °C: −8.6%	
		370–426 °C: −84.9%	
	Exo 455 °C combustion/degradation	426–500 °C: −4.7%	
M4	Endo 72 °C melting of PCL	-	−99.1%
	Endo 400 °C degradation	315–380 °C: −19.1%	
		380–427 °C: −78%	
	Exo 453 °C combustion/degradation	427–500 °C: −2%	
M5	Endo 72 °C melting of PCL	-	−98.3%
	Endo 153 °C melting of PLA	-	
	Endo 352 °C decomposition of PLA Endo 398 °C decomposition of PCL	270–315 °C: −4.9%	
		315–380 °C: −38.6%	
		380–420 °C: −50.8%	
	Exo 430 °C combustion	420–500 °C: −4%	
M6	Endo 71 °C melting of PCL	-	−98.4%
	Endo 154 °C melting of PLA	-	
	Endo 344 °C decomposition of PLA Endo 396 °C decomposition of PCL	270–315 °C: −6.4%	
		315–350 °C: −33.3%	
		350–420 °C: −57.9%	
	Exo 436 °C combustion/degradation	420–500 °C: −1.8%	

**Table 5:** Images of the water droplets on the scaffold’s surface and average contact angles.

Sample Code	Replicate 1	Replicate 2	Replicate 3	Average Contact Angle *
M1	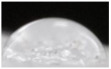	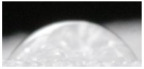	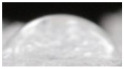	54.1 ± 6.5 ^a^
M2	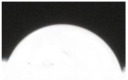	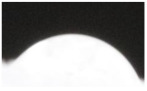	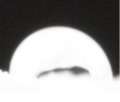	53.6 ± 7.9 ^a^
M3	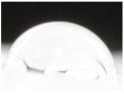	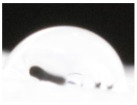	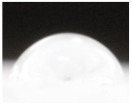	62.3 ± 5.3 ^a^
M4	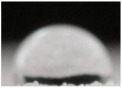	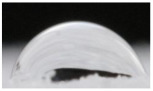	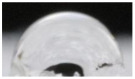	63.8 ± 17.8 ^a^
M5	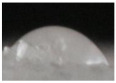	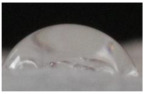	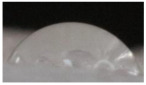	55.2 ± 5.6 ^a^
M6	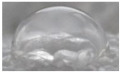	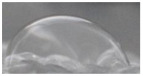	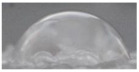	60.3 ± 5.8 ^a^

* The same letters mean that the differences are statistically nonsignificant according to one-way ANOVA/Tukey model.

**Table 6:** Macroscopic and microscopic images of the scaffolds after immersion in pig blood collected on citrate for 3 h at 37 °C.

Sample Code	Macroscopic Images		Microscopic Images (10×)
	Front View	Cross Section	
M1	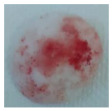	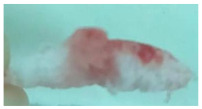	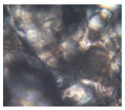
M2	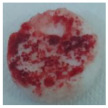	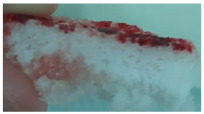	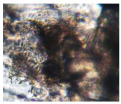
M3	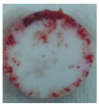	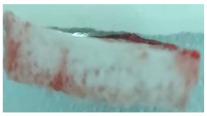	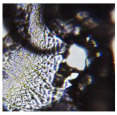
M4	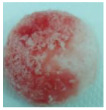	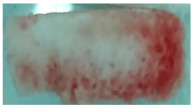	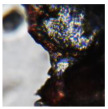
M5	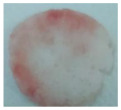	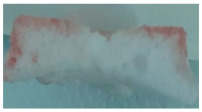	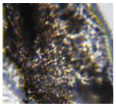
M6	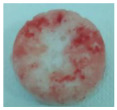	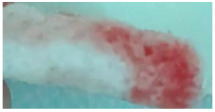	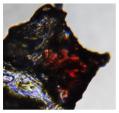

**Table 7:** Macroscopic images of the scaffolds with *Lactobacillus* 2 and 5 days after inoculation, and microscopic aspects of the bacterial culture.

Sample Code	After 2 Days		After 5 Days
	Macroscopic Image	Microscopic Image of the *Lactobacillus* Cell from the Scaffold Surface	
M1	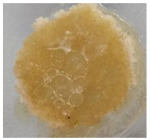	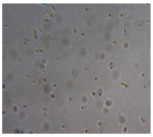	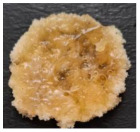
M2	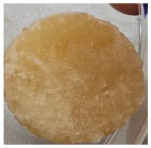	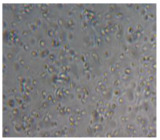	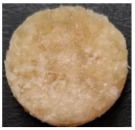
M3	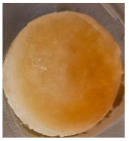	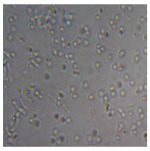	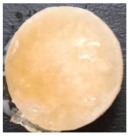
M4	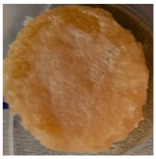	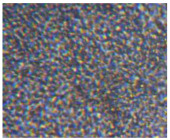	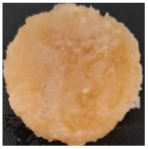
M5	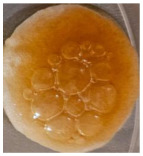	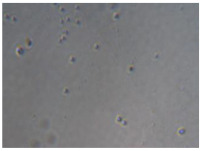	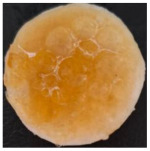
M6	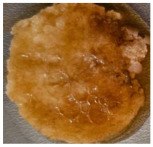	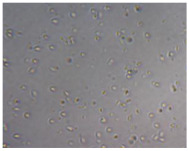	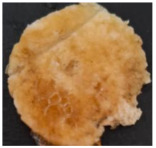

## Data Availability

The data presented in this study are available on request from the corresponding author—Prof. Dr. Anca Peter—pe-terancaluca@yahoo.com, from the security and privacy reasons.
